# Impact of Medical Treatment of Hemodynamically Significant Patent Ductus Arteriosus on Cerebral and Renal Tissue Oxygenation Measured by Near-Infrared Spectroscopy in Very Low-Birth-Weight Infants

**DOI:** 10.3390/medicina58040475

**Published:** 2022-03-25

**Authors:** Jūratė Navikienė, Arūnas Liubšys, Ernestas Viršilas, Tadas Žvirblis, Augustina Jankauskienė

**Affiliations:** 1 Faculty of Medicine, Institute of Clinical Medicine, Vilnius University, LT-08406 Vilnius, Lithuania; arunasliubsys@gmail.com (A.L.); ernestas.virsilas@santa.lt (E.V.); augustina.jankauskiene@santa.lt (A.J.); 2 Department of Mechanical and Material Engineering, Vilnius Gediminas Technical University, LT-10223 Vilnius, Lithuania; tadas.zvirblis@vilniustech.lt

**Keywords:** patent ductus arteriosus, preterm infant, cerebral oxygenation, renal oxygenation, near-infrared spectroscopy

## Abstract

*Background and objective:* Hemodynamically significant patent ductus arteriosus (hsPDA) can cause ductal steal and contribute to poor outcomes in preterm infants. Near-infrared spectroscopy (NIRS) allows us to continuously evaluate regional tissue oxygenation (rSpO_2_) and perfusion changes in underlying organs. The aim of this study was to evaluate the effect of medical treatment for hsPDA on cerebral and renal rSpO_2_ in infants less than 32 weeks of gestational age, and older than 72 h of life. *Materials and methods:* Infants with a gestational age of <32 weeks with hsPDA were prospectively studied before and during medical treatment. Two-site (cerebral and renal) rSpO_2_ monitoring by NIRS was performed 1 h before treatment (T0) and 24 h (T1), 24–48 h (T2), 48–72 h (T3) after the infusion of the first drug dose. *Results:* A total of 21 infants were studied. The mean day of life at treatment initiation was 8.2 (SD, 2.75). The DA diameter, LA/Ao ratio, and resistive index in the anterior cerebral artery (RI ACA) were significantly lower after treatment (*p* < 0.05). There were no significant differences in cerebral rSpO_2_, cerebral fractional tissue oxygen extraction (FTOE), and SpO_2_ comparing different time points. A significantly higher renal SpO_2_ value was recorded at T2 as compared with T0 (75.0%, SD 4.9%, vs. 69.4%, SD 7.6%; *p* < 0.013), while for renal FTOE, a tendency to lower values at T2 was observed (0.18, SD 0.05, vs. 0.24, SD 0.09; *p* = 0.068). *Conclusions*: Late (later than 7 days postpartum) hsPDA medical treatment with paracetamol or ibuprofen completely closed the duct only in a small proportion of preterm infants, despite a statistically significant reduction in the DA diameter, LA/Ao ratio, and RI ACA. Continuous renal, not cerebral, NIRS measurements can help to anticipate the efficacy of medical treatment of hsPDA in preterm infants. Large-scale prospective studies are needed to ascertain that renal and cerebral NIRS can be used as a reliable tool for evaluating the effectiveness of medical treatment for hsPDA.

## 1. Introduction

The patency of ductus arteriosus (PDA) is a frequent condition in preterm infants. As many as 60% to 70% of preterm infants of <28 week gestation receive medical or surgical treatment for a PDA [1].

Hemodynamically significant PDA (hsPDA) is associated with an apparent left-to-right shunt through the ductus, and can cause a so-called “ductal steal” phenomenon associated with reduced cerebral, renal, or mesenteric perfusion and increased pulmonary circulation. It leads to complications such as higher rate of respiratory failure and bronchopulmonary dysplasia (BPD), increased risk of intracranial hemorrhage (ICH), necrotizing enterocolitis (NEC), renal failure, and overall mortality [2]. Therefore, timely diagnosis and treatment of hsPDA is an important issue.

Neonatal echocardiography is the gold standard for the diagnosis of PDA and assessment of hemodynamic impact on the preterm infant’s circulation, and monitoring the therapeutic response [3]. Echocardiography is a valuable tool for the assessment of intracardiac changes due to PDA, i.e., DA size, shunt direction, Doppler flow pattern across the DA, volume overloading of left heart, left atrial-to-aortic root (LA/Ao) ratio, increased left pulmonary artery diastolic velocity, post-ductal aortic blood flow pattern, etc., but is less informative about regional blood flow changes in particular organs due to PDA [4,5]. Failure of circulation and/or oxygenation in vital organs, in fact, reflects the degree of clinical significance of PDA. Near-infrared spectroscopy (NIRS), one of the noninvasive tools widely used in neonatal clinical practice such as neonatal cardiac surgery and neonatal intensive care, allows the continuous evaluation of regional oxygenation (rSpO_2_) and perfusion changes in different organs (brain, kidney, liver, intestines), and can help arrive at an appropriate clinical decision on PDA treatment [6].

Regardless of numerous clinical studies on peripheral oxygenation/perfusion, there is still a lack of data on the impact of hsPDA on regional oxygenation of the most vulnerable organs in very low-birth-weight newborns, and existing reports are quite controversial. Therefore, the aim of this study was to evaluate the effect of medical treatment for hsPDA on cerebral and renal oxygenation monitored by NIRS in infants less than 32 weeks of gestational age and older than 72 h of life.

## 2. Materials and Methods

A prospective observational study was carried out in a tertiary-level neonatal intensive care unit (NICU) of the Neonatology Center, Vilnius University Hospital Santaros Klinikos, from November 2017 to June 2020. The study was approved by the Vilnius Regional Biomedical Research Ethics Committee (No. 158200-17-940-446, issued on 12 September 2017) and registered at clinicaltrials.com (reg. No NCT04295395). Informed parental consent was obtained before enrolment. Very low-birth-weight (<1500 g) infants with a gestational age of less than 32 weeks and older than 72 h of life who underwent echocardiographic screening for a PDA were included in the study. The exclusion criteria were major congenital anomalies, need for cardiovascular support with vasopressor medication, and culture-proven sepsis. Continuous two-site regional oxygenation monitoring using NIRS was performed 1 h before and during the 3-day hsPDA treatment course in infants with hsPDA.

According to the clinical protocol used in our NICU, the diagnosis of hsPDA requiring treatment was made by echocardiographic demonstration of a ductal left-to-right shunt, volume overload as a left atrial-to-aortic root (LA/Ao) ratio of ≥1.4, and DA size of >2 mm. All echocardiographic studies were performed by a pediatric cardiologist. The DA was evaluated from a high parasternal view using color Doppler assessment. The DA diameter was measured at the narrowest point. The LA/Ao ratio was measured in the parasternal long axis view using M-mode. The resistive index (RI) was evaluated in the anterior cerebral artery (ACA) using pulsed-wave Doppler on the day of NIRS monitoring before treatment. Echocardiography and ACA RI evaluation were carried out 1 h before and within a 24-h period after the last medication dose. Ibuprofen (10 mg/kg i.v. followed by 5 mg/kg, after 24 and 48 h; Pedea^®^, Orphan Europe, Paris, France) or paracetamol (15 mg/kg i.v. every 6 h for 3 days; Paracetamol Kabi^®^; Fresenius Kabi, Verona, Italy) was used for medical closure of PDA. The medications were infused continuously over a period of 15 min.

### 2.1. Data Collection

Perinatal and demographic data were collected, and cranial baseline ultrasound data were obtained. For two-site regional tissue oxygenation measurements, NIRS (NONIN SenSmart, X-100, Plymouth, MN, USA) was used. Neonatal/pediatric sensors (SenSmart 8004CB-NA non-adhesive with EQUANOXTM technology) were applied to the forehead lateral to the midline, superior to the eyebrow and inferior to the hairline, and on either the left or right posterior flank above the iliac crest and below the costal margin for measurements of cerebral and renal regional tissue oxygen saturation (rSpO_2_), respectively, using a wrap to secure the sensor. The NIRS device was left in place for continuous data acquisition. Recordings were briefly interrupted every 3 h to reposition the sensor medially or laterally to prevent skin bruising or breakdown. All NIRS data were recorded 1 h before treatment (T0) and 24 h (T1), 24–48 h (T3), and 48–72 h (T3) after the beginning of the first dose drug infusion. Simultaneously, SpO_2_ was measured to calculate the fractional tissue oxygen extraction (FTOE), which reflects the balance between tissue oxygen delivery and tissue oxygen consumption, indirectly reflecting tissue perfusion [7]. The saturation target was 89–95% with the lowest possible FiO_2_ and with lower and upper alarm limits of 88% and 96%, respectively. For each studied infant, data on the GA, birth weight, sex, type of delivery, antenatal steroids, Apgar scores at 1 and 5 min, surfactant administration, blood pH, lactate, platelet count, hemoglobin, hematocrit, blood pressure, FiO_2_ requirement at enrolment, need for respiratory support (noninvasive nasal continuous positive airway pressure or invasive [mechanical ventilation]), and urine output were collected. Arterial blood pressure was measured noninvasively on the right arm using a GE DINAMAP blood pressure monitor (GE Medical Systems Information Technologies, Milwaukee, WI, USA). The average of three measurements was recorded. The appropriate cuff sizes were chosen according to the manufacturer’s recommendations, with measurements performed on the right arm to ensure consistency. Measurements were only performed during routine care when the infant was in a quiet resting state, following minimal handling guidelines [8].

### 2.2. Statistical Analysis

Descriptive statistics such as frequency tables and mean (standard deviation) were used to describe quantitative and qualitative data, respectively. The paired *t* test was used to evaluate parameter differences before and after treatment. One-way ANOVA was used to evaluate mean differences when comparing more than two groups. Bonferroni correction was used for pairwise comparisons. A two-tailed p value less than 0.05 was considered to be significant. Statistical analysis was performed using Statistical Analysis System (SAS) package version 9.2.

## 3. Results

A total of 21 infants were examined. Their clinical characteristics and outcome data are presented in Table 1. The mean gestational age was 25.7 weeks (SD, 2.01), and birth weight was 810 g (SD, 219). Mean day of life of treatment initiation was 8.2 (SD, 2.75). Thirteen patients (61.9%) received paracetamol, while others were treated with ibuprofen. DA closure was achieved in four infants. Sixteen infants had DA diameter decreased.

All echocardiographic parameters before and after treatment are presented in Table 2. The DA diameter, LA/Ao ratio, and RI ACA were significantly lower after treatment. No effect on the DA diameter or shunt was observed for one patient. There was a significant increase in blood pressure after treatment. Urine output was not affected by the therapy (*p* = 0.11).

NIRS and SpO_2_ measurements at different time points are presented in Table 3. There were no significant differences in cerebral rSpO_2_, cerebral FTOE, or SpO_2_ comparing different time points. We found higher renal rSpO_2_ values at T2 compared to the baseline values (T0). However, this improvement diminished at T3. Renal FTOE values were lower at T2 than T0, but not statistically significant.

## 4. Discussion

In this study, 21 very low-birth-weight infants with hsPDA were treated with paracetamol or ibuprofen for ductus closure. Although a positive effect of treatment was achieved in a majority of the patients, complete closure of the ductus occurred only in four patients. Issues such as when to start medical treatment and what are the most appropriate medication and the initial dose still remain controversial [9,10,11]. Studies have shown that PDA treatment during the first week of life is associated with a higher rate of successful ductus closure compared with later treatment [12,13]. PDA treatment with ibuprofen with the standard dosing scheme of 10–5–5 mg in older neonates is less successful [14]. It is also known that a double dose of ibuprofen closes the PDA more frequently than the usual dose, without increasing the risk of side effects [14,15]. Several systematic reviews and meta-analyses have shown that oral administration of ibuprofen is associated with greater success in closing the PDA than intravenous administration [16,17]. Nevertheless, a recent systematic review and meta-analysis showed that 38% of PDA closure occurs in preterm babies without any treatment [16]. Some authors reported even higher rates of spontaneous closure of PDA (up to 78–98%) in infants >28 weeks gestation and >1000 g birth weight [18,19]. Therefore, delayed medical treatment after 7 to 10 days of life and just for those babies with hsPDA, or even a conservative approach (without any medical treatment), is recommended more often as compared with prophylactic PDA treatment [20].

Several systematic reviews and meta-analyses [21,22,23,24] have shown a similar effect of paracetamol and ibuprofen in PDA closure; therefore, we did not analyze the effect of treatment of each medication separately. On the other hand, paracetamol is used more often in our daily practice due to its fewer side effects (fewer cases of renal failure and gastrointestinal bleeding as well as lower serum bilirubin and creatinine levels) [21,22] and a lower price compared with intravenous ibuprofen. In our study, medical treatment of hsPDA was initiated late (mean day of life at treatment initiation was 8.2), and we used a standard dose of intravenous ibuprofen and paracetamol; therefore, we can speculate that all these factors may have contributed to a low rate of complete closure of PDA in our patients.

Despite a low closure rate, the DA diameter and LA/Ao ratio decreased significantly after medical treatment in a majority of the patients. It is known that late medical treatment for symptomatic patent ductus is less effective than early (targeted or prophylactic) treatment; however, it reduces the DA diameter and the need for surgical ligation of the PDA [25]. El-Khuffash et al. found that late administration of intravenous paracetamol was associated with only 25% of successful ductal closures, but it significantly reduced the need for surgical treatment of PDA [26].

Measuring the RI in cerebral arteries, especially in the ACA, is one of the most commonly used and simplest methods for assessing cerebral blood flow in both term and preterm infants. PDA is considered to be a usual cause for elevated RI in preterm infants [27,28]. In line with our study, Pees et al. found a significant decrease in IR ACA in premature infants with hsPDA after treatment with ibuprofen [29].

Another finding of our study was a significant increase in systolic and diastolic blood pressure after medical hsPDA treatment. According to Rios et al., hsPDA can be associated with circulatory insufficiency and hypotension, and medical treatment can normalize the blood flow and blood pressure of a preterm infant [30]. This was confirmed by other authors who studied the effect of medical treatment on the blood pressure of preterm infants in the presence of PDA [31,32].

HsPDA is known to cause regional oxygenation and circulatory changes in vital organs such as the brain and kidneys [33,34]. In this study, we made the assumption that if medical treatment reduced PDA-induced hemodynamic changes, it could also significantly improve regional brain and kidney oxygenation and blood flow. On the other hand, regional improvements in brain and kidney oxygenation and blood flow could be used for evaluating the success of PDA treatment. Our study showed significant improvement only in renal SpO_2,_ and a tendency to improve renal FTOE after 24–48 h from the initiation of medical treatment, although this effect disappeared after 72 h. Meanwhile, treatment had no effect on cerebral oxygenation and regional blood flow, despite there being a statistically significant reduction in RI ACA after treatment, reflecting better cerebral blood flow.

Some clinical studies demonstrated a faster and marked deterioration of renal oxygenation and blood flow in the presence of hsPDA, comparing with no effect or less apparent effect on brain oxygenation and perfusion [35,36]; therefore, it is likely that medical treatment-related closure or constriction of PDA primarily improves renal oxygenation and blood circulation. Moreover, the ductal shunt occurs predominantly from the lower rather than the upper body circulation [37]. Cerebral rSpO_2_ is primarily affected by pre-ductal systemic perfusion and cerebral oxygen extraction, while renal oxygenation is impacted by post-ductal diastolic steal [38].

Contrary to our study, some studies demonstrated increased cerebral SpO_2_ and reduced FTOE after successful PDA closure [39,40], while others showed an increase only in FTOE without any effect on rSpO_2_ [7]. In line with these studies, Poon et al. recently documented significant improvement in cerebral SpO_2_ and FTOE after medical closure of PDA, but in contrast to our study, they did not find significant changes in renal rSpO_2_ after closure of PDA. They also did not observe any correlation between cerebral rSpO_2_ and renal rSpO_2_ and PDA size [40]. Dani et al. found no changes in cerebral oxygenation after treatment of PDA with paracetamol and ibuprofen [41]. Such contradictory research data obtained by different authors do not allow us to draw definite conclusions about the impact of medical treatment for hsPDA on the changes in regional oxygenation and blood circulation in a newborn’s kidney and brain. These differences may be caused by a number of factors, such as different age and weight of infants enrolled in the studies, different PDA diameter before treatment, different time of initiation of the treatment, and different medications and doses used. For most of the patients included in our study, the PDA did not close completely but only narrowed, and this could have resulted in treatment not affecting cerebral SpO_2_ and cerebral FTOE. It can be assumed that if the PDA were completely closed for most of our patients, the effect of medical treatment on cerebral oxygenation and blood circulation would be more apparent. On the other hand, even narrowing of PDA related to medical treatment of hsPDA had an effect on renal oxygenation and blood circulation detected by continuous NIRS. Our findings suggest that renal rSpO_2_ but not cerebral rSpO_2_ measured by continuous NIRS could help us anticipate the effectiveness of medical treatment for hsPDA in preterm infants.

Absence of changes in brain oxygenation and blood flow after medical treatment of PDA might be directly related to the failure of ductal closure. A relatively small sample size of the study population did not allow revealing more clear associations between PDA closure or constriction after medical treatment as well as cerebral and renal oxygenation and regional circulation. This could be due to the study being statistically underpowered. Other limitations were the design of a single-site observational trial without a control arm, and the usage of different medications for treatment.

## 5. Conclusions

Late (later than 7 days postpartum) medical treatment of hsPDA with paracetamol or ibuprofen completely closed the duct only in a small proportion of preterm infants, despite a statistically significant reduction in the DA diameter, LA/Ao ratio, and RI ACA. Continuous renal, not cerebral, NIRS measurements can help anticipate the efficacy of medical treatment for hsPDA in preterm infants. Large-scale prospective studies are needed to ascertain that renal and cerebral NIRS can be used as a reliable tool for the evaluation of the effectiveness of medical treatment for hsPDA.

## Figures and Tables

**Table 1:** Clinical characteristics and outcome data.

Characteristic	Value
Gestation age, mean (SD), weeks	25.7 (2.0)
Birth weight, mean (SD), g	810 (219)
Male, *n* (%)	12 (57.1)
Apgar score at 1 min, mean (SD)	5.9 (2.1)
Apgar score at 5 min, mean (SD)	7.3 (1.3)
Day of life at measurement, mean (SD)	8.2 (2.8)
Hematocrit, mean (SD), %	39.0 (7.2)
HB, mean (SD), g/L	132.7 (26.3)
Platelets, mean (SD), ×10^9^/L	274.1 (153.4)
Antenatal steroids 2×, *n* (%)	16 (76.2)
Chorioamnionitis, *n* (%)	6 (28.6)
Caesarean section, *n* (%)	11 (52.4)
Surfactant, *n* (%)	20 (95.2)
Mechanical ventilation, *n* (%)	19 (90.5)
Noninvasive respiratory support, *n* (%)	1 (4.8)
FiO_2_, mean (SD)	0.32 (0.09)
IVH (III–IV), *n* (%)	7 (33.3)
Ibuprofen, *n* (%)	8 (38.1)
Paracetamol, *n* (%)	13 (61.9)
Treatment outcome, *n* (%) Closure Constriction Failure	4 (19.0) 16 (76.2) 1 (4.8)

HB—hemoglobin; IVH—intraventricular hemorrhage.

**Table 2:** Echocardiographic parameters before and after treatment.

Parameter	Before Treatment	After Treatment	*p* Value
DA diameter, cm	0.25 (0.04)	0.16 (0.09)	<0.001
LA/Ao ratio	1.69 (0.19)	1.41 (0.24)	<0.001
Urine output, mL/kg/h	3.46 (0.89)	3.89 (1.11)	0.111
RI ACA	0.85 (0.14)	0.75 (0.08)	0.018
Systolic BP, mm Hg	52.7 (6.1)	59.0 (8.1)	0.002
Diastolic BP, mm Hg	24.7 (6.0)	32.0 (6.2)	<0.001
Mean BP, mm Hg	32.8 (6.0)	40.0 (6.3)	<0.001

Values are mean (SD). DA—ductus arteriosus; LA/Ao—left atrial-to-aortic root; RI ACA—resistive index in the anterior cerebral artery; BP—blood pressure.

**Table 3:** NIRS and SpO_2_ measurements at different time points.

Parameter	T(0)	T(1)	T(2)	T(3)	*p* Value
Cerebral rSpO_2_, %	77.4 (4.5)	77.2 (3.6)	77.9 (2.8)	78.1 (3.4)	0.879
Renal rSpO_2_, %	69.4 (7.6) *	73.3 (4.2)	75.0 (4.9) *	73.9 (5.3)	0.013
SpO_2_, %	92.2 (2.0)	92.7 (1.1)	92.7 (1.2)	92.9 (1.2)	0.519
Cerebral FTOE	0.16 (0.05)	0.17 (0.04)	0.16 (0.03)	0.16 (0.04)	0.721
Renal FTOE	0.24 (0.09)	0.20 (0.04)	0.18 (0.05)	0.20 (0.06)	0.068

Values are mean (SD). FTOE—fractional tissue oxygen extraction. * Statistically significant difference between T0 and T2 time points (post hoc analysis for pairwise comparisons).

## Data Availability

The data presented in this study are available on reasonable request from the corresponding author.
